# Nutrient Intake and Status in Children and Adolescents Consuming Plant-Based Diets Compared to Meat-Eaters: A Systematic Review

**DOI:** 10.3390/nu15204341

**Published:** 2023-10-11

**Authors:** Nicole Neufingerl, Ans Eilander

**Affiliations:** Unilever Foods Innovation Centre, 6708 WH Wageningen, The Netherlands; nicole.neufingerl@unilever.com

**Keywords:** plant-based diet, dietary intake, micronutrients, nutritional status, vegetarian, vegan, children, adolescents

## Abstract

Health authorities increasingly recommend sustainable and healthy diets rich in plant foods and with moderate amounts of animal foods. However, there are concerns about whether such diets can meet all nutrient requirements, especially in children and adolescents, who have relatively high nutrient needs for growth and development. Therefore, we aimed to evaluate the nutrient intake and status of children and adolescents (2–18 y) consuming plant-based (i.e., vegetarian and vegan) diets compared to those of meat-eating children following a systematic literature review of studies published between 2000 and 2022. Mean intake and status data of nutrients were calculated across studies and benchmarked to dietary reference values and cut-off values for nutrient deficiencies. A total of 30 studies were included (15 in children 2–5 y, 24 in children 6–12 y, and 11 in adolescents 13–18 y). In all diets, there were risks of inadequate intakes of vitamin D and calcium. Children consuming meat had a risk of inadequate folate and vitamin E intake; and mean fiber, SAFA, and PUFA intakes were not in line with the recommendations. Children consuming plant-based diets risked inadequate vitamin B12, iron, and zinc intakes. In contrast to vegans, vegetarian children may not meet the recommended intakes of fiber, SAFA, and possibly PUFA, but their mean intakes were more favorable than in meat-eating children. Although the data are limited and need further validation, our findings indicate that there are risks of nutritional inadequacies in all diet groups. Therefore, increasing consumption of a variety of plant-based foods, in combination with food fortification and supplementation where needed, is recommended for children and adolescents to have sustainable and nutritionally adequate diets.

## 1. Introduction

Globally and locally, health authorities increasingly recognize the need for healthy and sustainable diets that are rich in fruits, vegetables, pulses, whole grains, and nuts, with some fish, eggs, poultry, and dairy but limited red meat and starchy vegetables [1,2].

Although well-planned plant-based diets that are adequately supplemented are considered healthful for both adults and children [3,4], there is also debate about whether the exclusion of animal foods from the diet may put population groups with specific nutritional requirements at risk [5,6]. Children and adolescents are among these vulnerable groups, as they have relatively high nutritional needs for growth and development [3,4,5,6]. Meat and dairy are protein-dense foods with high protein quality, containing all indispensable amino acids to support physiological functions as well as growth and development [7]. Plant proteins usually have lower protein quality due to the lower digestibility of amino acids from plant foods. In addition, plant proteins usually fall short in one or more indispensable amino acids, though this can be compensated by eating a varied diet, as a shortfall in a specific amino acid in one food can be compensated for by a relative surplus of the same amino acid in another food [8]. In addition, animal foods are good sources of iron, zinc, selenium, calcium, riboflavin, and vitamins A and B12, and fish and seafood in particular are naturally good sources of vitamin D, iodine, and the long-chain omega-3 fatty acids EPA and DHA [5,6]. On the other hand, plant foods are high in fiber and vitamins C and E, and plant oils are good sources of polyunsaturated fatty acids (PUFA), particularly the essential fatty acids α-linolenic acid (ALA) and linoleic acid (LA), which are important for the growth and development of children [9,10]. Although the bioavailability of certain nutrients may be limited in predominantly plant-based diets, and particularly in vegan diets [5,6], it has been suggested that plant-based diets that are sufficient in energy and contain a variety of nutrient-dense plant foods can provide the dietary requirements of protein and most micronutrients [3,4].

Only a few studies have investigated whether plant-based diets are nutritionally adequate for growing children and adolescents. A systematic review by Sutter et al. showed that there is a high risk of deficiencies of calcium and vitamins D and B12 in children when vegan diets are poorly planned and indicated that for micronutrients like iodine and iron more data are needed [11]. Schürmann et al. evaluated results from 16 studies conducted between 1988 and 2013 in 0–18-year-old children and concluded that the intake of folate, vitamin C, and dietary fiber was relatively high in vegetarians compared to reference values and/or control groups [12]. In this review, it was found that a low status of vitamin B12 was reported in one study and a low status of vitamin D in two studies.

Micronutrient deficiencies are still widely prevalent among children and adolescents, with an estimated 50% of pre-school children having at least one micronutrient deficiency [13]. In addition, undernutrition remains a large problem, with 22% of children under 5 years of age being stunted [14], whereas overnutrition rates are increasing, with more than 20% of children and adolescents being overweight in 2020 [15]. Several literature reviews have indicated that children and adolescents consuming vegetarian diets have a healthier weight and possibly healthier blood lipid profile compared to children on omnivorous diets [12,16] and that vegan children had a normal weight and were less often obese [11].

Given the current gaps in literature regarding the nutritional adequacy of children consuming plant-based diets, this systematic review aims to evaluate and compare the nutrient intake and status of children and adolescents (2–18 y) consuming plant-based (i.e., vegetarian and vegan) diets with their meat-eating counterparts.

## 2. Materials and Methods

### 2.1. Search Strategy

We used a systematic approach to identify studies reporting energy and nutrient intake and/or status of children and adolescents consuming plant-based diets, including studies that compared these data with children and adolescents consuming meat-containing diets. The literature search for this review was part of a broader search strategy, which was originally not limited to children and adolescents but included the general adult population. Results of the review in the general adult population have been published separately [17]. The search string included different terms for plant-based diets, in combination with terms on dietary intake or nutritional status, along with predefined nutrients of specific interest, i.e., (diet OR intake OR “nutritional status” OR adequacy OR deficien*) in the title or abstract AND (vegetarian OR pescatarian OR vegan OR flexitarian OR meat?free OR “less meat” OR no?meat OR dairy?free OR no?dairy OR plant?based OR plant?forward OR sustainable) in the title or abstract AND (nutrient* OR vitamin* OR mineral* OR micronutrient* OR zinc OR iodine OR iron OR calcium OR thiamin? OR riboflavin OR niacin OR “pantothenic acid” OR pyridoxin OR biotin OR “folic acid” OR folate OR cobalamin OR retinol OR caroten* OR “omega-3 fatty acid” OR “fish fatty acid*” OR PUFA OR “polyunsaturated fatty acid*” OR DHA OR “docosahexaenoic acid” OR “eicosapentaenoic acid” OR EPA OR an?emi*) in all fields. The initial literature search was conducted in the PubMed database and was limited to articles published from 2000 onwards and in the English language. Older articles were not included, as they were not considered representative for current plant-based dietary patterns due to developments in the availability and range of plant-based products in recent decades. The initial search took place in July 2021. In October 2022, we carried out a one-time update of this search, limited to articles published from January 2021 onwards and narrowed down to children and adolescents. To this end, the original search string was expanded with the following terms: (child* OR girl OR girls OR boy OR boys OR youth OR adolescent OR adolescents OR school OR student OR students OR teenage* OR teens OR toddler OR toddlers) in the title or abstract.

Reference lists of relevant (systematic) reviews and meta-analyses obtained from the literature search were checked for additional pertinent studies that were published from 2000 onwards. For the reporting of this systematic review, the Preferred Reporting Items for Systematic reviews and Meta-Analyses (PRISMA) was used.

### 2.2. Inclusion and Exclusion Criteria

- Study population: Generally healthy children and/or adolescents aged 2 to 18 years. We excluded studies on infants (<2 years) because they have specific dietary needs, including breastfeeding and complementary foods. We also excluded studies conducted in populations with specific diseases.
- Type of studies: Observational studies and intervention studies (baseline data only), that compared nutrient intake and/or status of participants following a predominantly plant-based diet with participants following a conventional diet with meat were included. In addition, studies that reported only on subjects following a predominantly plant-based diet were also included. Generic reviews, case studies, and articles not published in the English language were excluded.
- Diets: To be included in our review, studies had to report on voluntary self-selected diets with a primary focus on reducing animal food intake. Studies reporting on imposed or predesigned plant-based diets (e.g., marginal plant-based staple diets in developing countries, a prescribed vegetarian diet intervention, or a modeled vegetarian diet scenario) were excluded, as well as articles on overly restrictive plant-based diets (e.g., raw food diet, macrobiotic diet) or healthy diets designed to lower non-communicable diseases (e.g., DASH diet, Mediterranean diet).
- Outcome parameters: The included studies provided data on one or more of the following parameters: dietary intake of energy, protein, saturated fatty acids (SAFA), poly-unsaturated fatty acids (PUFA), α-linolenic acid (ALA), and eicosapentanoic acid (EPA); docosahexanoic acid (DHA); dietary intake or nutritional status of micronutrients; bone markers.

### 2.3. Data Extraction

The identified articles were exported to an Endnote library and duplicates were removed. For each included study, we extracted information about population characteristics (age, gender), study location (country), reported diet patterns, inclusion/exclusion of supplement users, and publication date. For each dietary pattern, we extracted means, standard deviations (SD) or standard errors (SE), medians, percentiles or ranges of parameters of dietary intake, and nutritional status of the following nutrients: energy intake, protein, SAFA, PUFA, total n-3 fatty acids, ALA, EPA, DHA, fiber, vitamin A, B1, B2, B6, B12, niacin, folate, vitamin C, D, E, iron, zinc, calcium, iodine, magnesium, and phosphorus. We also extracted data on the prevalence of inadequate intake and the prevalence of deficiencies of these nutrients and their corresponding cut-off criteria. In addition, data on hemoglobin, anemia, and bone markers were collected for evaluation of iron and calcium status.

### 2.4. Data Handling

The definition and naming of vegetarian, vegan, and other types of plant-based diets varied across studies. To ensure a consistent interpretation of the data, we applied the following uniform definitions to categorize all reported dietary patterns:

- Vegan: consuming no meat, fish, dairy, and eggs at all/not during the days of dietary assessment OR ≤ once per month OR self-defined vegans;
- Vegetarian: consuming no meat and fish at all/not during the days of dietary assessment OR ≤ once per month OR self-defined vegetarians;
- Pesco-vegetarian: consuming no meat at all/not during the days of dietary assessment OR ≤ once per month OR self-defined;
- Semi-vegetarian: consuming meat (and fish) ≤ once per week but > once per month OR consuming meat (and fish) “seldom”/“occasionally”;
- Meat eating: consuming meat > once per week OR self-defined.

Some studies did not make a distinction between vegetarians and vegans or between pesco-vegetarians and vegetarians, or they reported combined values for these groups. In these cases, the diets were categorized as “vegetarian”. If a study reported separate data for different subgroups (e.g., based on age, gender, supplement use), the data were recorded separately for each subgroup.

Studies that did not mention that intake from supplements were considered were classified as reporting nutrient intake from foods alone.

### 2.5. Data Analysis

The primary outcomes of our review are the aggregated descriptive data on nutrient intake and status. For ease of comparison and further analysis, nutrient intake and status data that were reported as medians, percentiles, SE or ranges were converted into means and SD using standard formulas [18,19]. Average nutrient intakes and status for the different dietary patterns were then calculated across all studies. If a study reported data for different age or gender groups, they were treated as individual data points in these calculations. For micronutrient intake, separate analyses were conducted for studies that assessed intake from foods alone and for studies that assessed intake from foods and supplements. Studies that did not mention that intake from supplements were classified as reporting nutrient intake from foods alone.

Secondary outcomes are the number of studies with average intakes below dietary reference values (DRVs), and the number of studies that did or did not find a significant difference in nutrient intake or status between dietary patterns. Per individual study, we compared the mean nutrient intakes of the different dietary patterns with the DRVs and reported the number of studies where mean intakes were above or below the reference values. Micronutrient intakes were compared with the estimated average requirement(s) (EAR) of the respective age and gender group(s) that were included in the study [20,21]. Because of the lower bioavailability of iron and zinc from predominantly plant-based diets, iron and zinc intakes of vegans and vegetarians were compared to a bioavailability-adjusted EAR, reflecting increased requirements according to the recommendation of the IOM [20]. Average protein, PUFA, and ALA intakes were compared with the lower level of the acceptable micronutrient distribution range (L-AMDR), average SAFA intakes with the upper level of the acceptable micronutrient distribution range (U-AMDR), and average EPA and DHA, and fiber intakes were compared with adequate intakes (AIs) [9,10]. DRVs often differ for different age groups, as well as for girls and boys. Therefore, when a study reported intake data across different age groups, intakes were compared against the dietary reference values that correspond to the mean or median age of the children. When intake data were reported for girls and boys combined, intakes were compared against the average of the dietary reference values for boys and girls. Average nutrient status of the different dietary patterns was compared to cut-off levels indicating deficiency, as defined by the World Health Organization (WHO) or the Institute of Medicine (IOM). Furthermore, per individual study, we assessed whether significant differences between dietary patterns were observed regarding nutrient intake, status, prevalence of inadequate intakes, or prevalence of nutrient deficiencies.

For reasons of consistency and comparability, we only report on prevalence numbers of inadequate nutrient intakes and nutrient deficiencies if appropriate biomarkers and cut-offs, according to the definitions of the WHO or IOM, were used.

Finally, we conducted a systematic assessment of the risk of bias for all included studies based on the Observational Study Quality Evaluation (OSQE) checklist [22]. The OSQE considers the following aspects: the representativeness of the study sample, the validity of the exposure variable and of the outcome variables, the justification of the sample size, a priori determined analysis, controlling of confounders, and potential conflicts of interest.

## 3. Results

### 3.1. Study Selection and Study Characteristics

**Figure 1:**
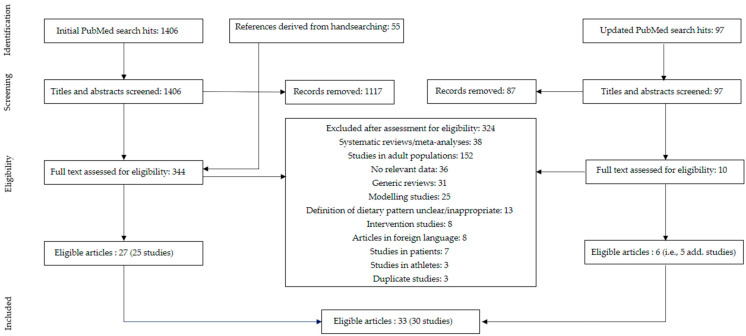
Flow diagram.

The initial literature search retrieved 1406 hits from PubMed; 55 additional references were identified by handsearching reference lists of relevant reviews and meta-analyses. After screening titles and abstracts of all search results and reading 344 articles in full, we identified 27 relevant articles (i.e., 25 relevant studies) in children and adolescents. The updated literature search in 2022 resulted in 97 additional hits. Handsearching references from newly identified reviews did not deliver extra hits. After screening titles and abstracts, ten articles were read in full to assess their eligibility, of which six articles (i.e., five additional studies) were included. Altogether, this led to a total of 33 articles reporting on 30 individual studies that were included in this review. See Figure 1 for more details of the screening process.

In 6 out of the 30 studies, some of the subjects were younger than two years [23,24,25,26,27,28]. Yet, these studies were included because the overall age range, or the reported mean/median age, suggested that the majority of children were older than two years.

Twenty studies reported on nutrient intake data, most of which assessed nutrient intake from foods alone. Two studies reported nutrient intake from foods alone as well as intake from foods and supplements combined [27,29]. Twenty-three studies reported on nutrient status, of which three excluded supplement users. Most studies (n = 23) compared vegetarian and meat-eating dietary patterns. Nine of these studies reported data on vegetarians, vegans, and/or pesco-vegetarians combined. However, most of the subjects in these studies followed a vegetarian diet, and therefore the diets were categorized as “vegetarian”. No study reported on pesco-vegetarian children or adolescents as a separate dietary group.

In three studies, we classified dietary patterns as “semi-vegetarian”. One of these studies reported on a dietary pattern in India defined by principal component analysis, which was characterized among others by limited consumption of meat (i.e., chicken and mutton were, on average, consumed 2 times per month) [30]. In another study from the USA, subjects had classified themselves as being vegetarian or vegan, but after questioning, 62% indicated that they did consume chicken and/or fish. As this study presented data for true vegetarians and vegans and for semi-vegetarians combined, and because most children followed a semi-vegetarian diet, the diet was classified as “semi-vegetarian” [31]. In the third study, semi-vegetarian children reported to consume meat and fish less than once a week [32].

Based on the risk of bias assessment, the quality of the included studies was mixed, with 53% of studies scoring well on at least five out of eight quality criteria and 47% of studies scoring well on less than five criteria (see Supplementary Material Table S6). Most studies were conducted in Europe. For an overview of the characteristics of the included studies, see Table 1; an overview of the details of each included study can be found in Supplementary Table S1.

### 3.2. Energy, Protein, Fatty Acids, and Fiber

#### 3.2.1. Energy

Sixteen studies reported on energy intake. None of the 13 studies that directly compared energy intake across dietary patterns found a significant difference between energy intake of meat eaters, (semi-)vegetarians, or vegans. For summary statistics on energy and nutrient intakes, see Supplementary Material Table S2.

#### 3.2.2. Protein

**Figure 2:**
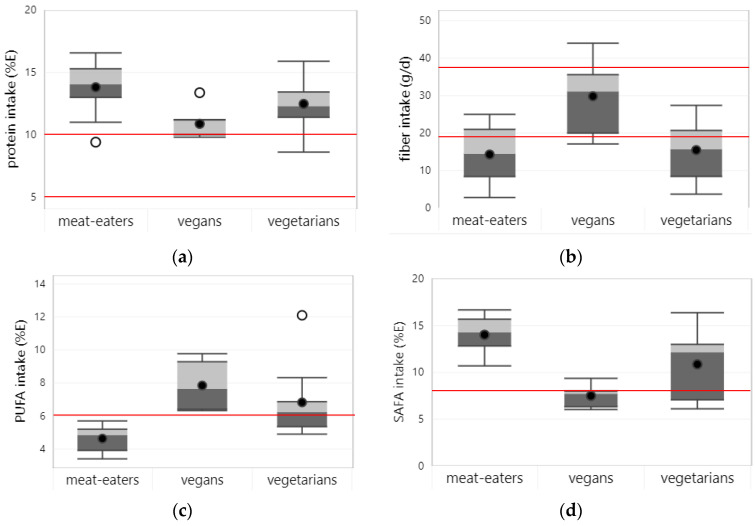
Boxplots represent 25th, 50th, and 75th percentiles of intake with whiskers at the < 1.5 interquartile range (IQR) per dietary pattern. Black dots represent mean intake and white dots outliers with >1.5 IQR: (**a**) protein; (**b**) fiber; (**c**) polyunsaturated fatty acids (PUFA); (**d**) saturated fatty acids (SAFA). Straight red lines represent the lower level of the acceptable macronutrient distribution range (AMDR) for protein (i.e., 5%E for children ≤ 3 y; 10%E for children > 3 y) and PUFA (i.e., 6%E) and the upper level of the AMDR for SAFA (i.e., 8%E). For fiber, the adequate intake (AI) for the age and gender groups with the lowest (19 g/d) and highest (38 g/d) dietary requirements that were included in the studies is shown. Boxplots are only shown for macronutrients, for which intake data from at least 3 studies were available for at least one of the dietary patterns.

Seventeen studies reported on protein intake in children and adolescents. Across studies, average protein intake in vegans (10.9%E) and vegetarians (12.5%E) was lower compared to meat eaters (13.8%E) (see Figure 2a). Yet, protein intake mostly met and exceeded the L-AMDR of the respective age and gender groups [10]. One study (out of 14) in vegetarian and meat-eating children from Poland [33] and one study (out of 4) among vegan children from Ghana [23] reported protein intakes below the L-AMDR.

Fifteen studies compared protein intake between dietary patterns, of which nine showed a significantly lower intake in vegetarians and/or vegans compared to meat-eating children and adolescents. The other studies did not show a significant difference in protein intake between diet groups. One study that reported on semi-vegetarian children and adolescents from the USA reported a protein intake similar to that of meat eaters (13.7%E) [31].

A study among 4–9-year-old vegetarian children in Poland found that 2% of children had intakes below the L-AMDR (<10%E) [34].

#### 3.2.3. Fiber

Thirteen studies reported on fiber intake of children and adolescents. Across studies, average fiber intake was clearly higher in vegans (29.8 g/d) than in vegetarians (15.5 g/d) and meat eaters (14.3 g/d). In all studies, fiber intake of meat eaters was below the AI for the respective age and gender groups [10]. In addition, vegetarians’ fiber intake was below the AI in most (i.e., 8 out of 10) studies, whereas vegans had a fiber intake above the AI in most (i.e., 4 out of 5) studies. Among the studies that directly compared fiber intake between dietary patterns (n = 11), all but one found a significantly higher intake in vegans and (semi-)vegetarians compared to meat eaters.

#### 3.2.4. Polyunsaturated Fatty Acids

Nine studies reported on PUFA intake, with, on average, a higher PUFA intake in vegans (8.0%E) and vegetarians (6.8%E) than in meat-eating (4.6%E) children and adolescents (see Figure 2b). Meat eaters failed to meet the L-AMDR for PUFA (i.e., 6%E [9]) in all studies (i.e., 7/7 studies) and vegetarians in three out of seven studies. Vegans’ PUFA intake was within the AMDR, based on four available studies. The highest PUFA intake (i.e., 12.1%E) was seen in a study in vegetarian adolescents in China [35].

Five out of seven studies that compared PUFA intakes between dietary patterns found that vegetarians and vegans had higher PUFA intakes than meat eaters. The other two studies found no significant difference in PUFA intake between vegetarians or semi-vegetarians and meat eaters [26,31].

No studies reported on the prevalence of children with PUFA intakes below the L-AMDR.

#### 3.2.5. N-3 Fatty Acids

Two studies reported on the intake of n-3 fatty acids in children in Finland and Germany [27,29]. Both studies reported on intakes from foods, as well as from foods and supplements, with very similar results. Overall, EPA and DHA intakes were highest in meat eaters. In the Finnish study, EPA and DHA intake of meat-eating and vegetarian children met the AI (i.e., 100 mg/d), yet vegans’ EPA and DHA intake fell short [27]. In the German study, EPA and DHA intakes were below the AI in vegan, vegetarian, and meat-eating children [29]. Both studies showed a significant difference in EPA and DHA intake between meat eaters and vegans; the difference between vegetarians and meat eaters was only significant in the German study. Furthermore, both studies found that ALA intake was significantly higher in vegans and vegetarians compared to meat eaters, with the highest intake in vegans. ALA intake was above the L-AMDR (i.e., 0.5%E) in all dietary patterns.

One Polish study assessed serum EPA concentrations in children and adolescents and found no significant difference between vegetarians and meat eaters [36].

#### 3.2.6. Saturated Fatty Acids

Eight studies reported on intake of SAFA, with, on average, the highest intakes in meat-eating (14.0 %E) followed by vegetarian (10.9% E) and vegan (7.5% E) children and adolescents (see Figure 2c). Among meat eaters and (semi-)vegetarians, SAFA intakes exceeded the U-AMDR (i.e., 8% E) in all (i.e., 7/7) or most (i.e., 6/7) studies. Yet, SAFA intake of vegan children and adolescents was in line with the AMDR in most (i.e., 4/5) studies.

Among studies that compared SAFA intake between dietary patterns (n = 7), all but one found significantly lower intakes in vegetarians and vegans compared to meat-eating children and adolescents. Furthermore, two studies in New Zealand and the USA found that the percentage of children and adolescents whose SAFA intake exceeded the DRV was significantly higher among meat-eating compared to (semi-)vegetarian children and adolescents [31,37].

### 3.3. Vitamins

#### 3.3.1. Vitamin A

**Figure 3:**
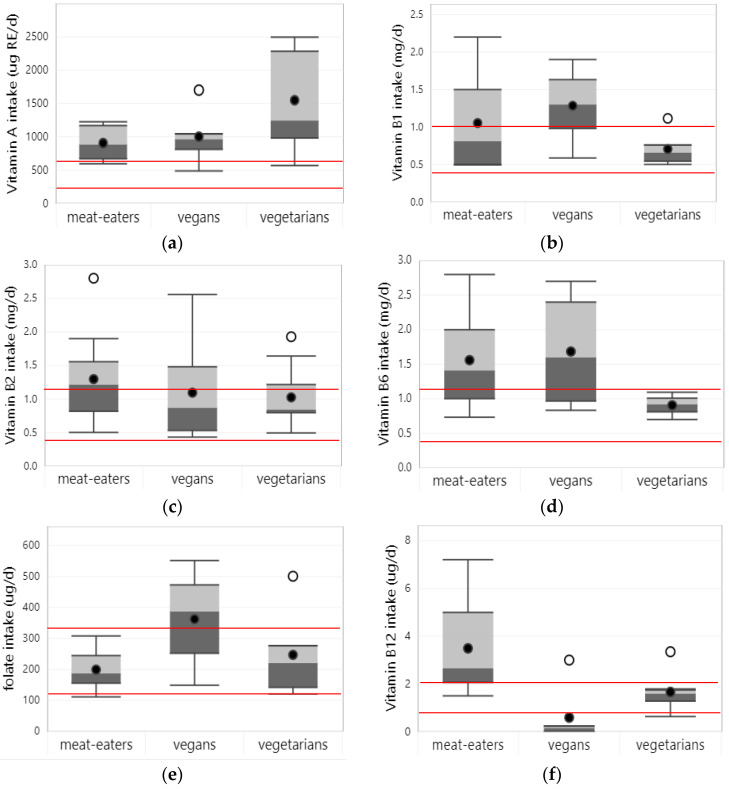

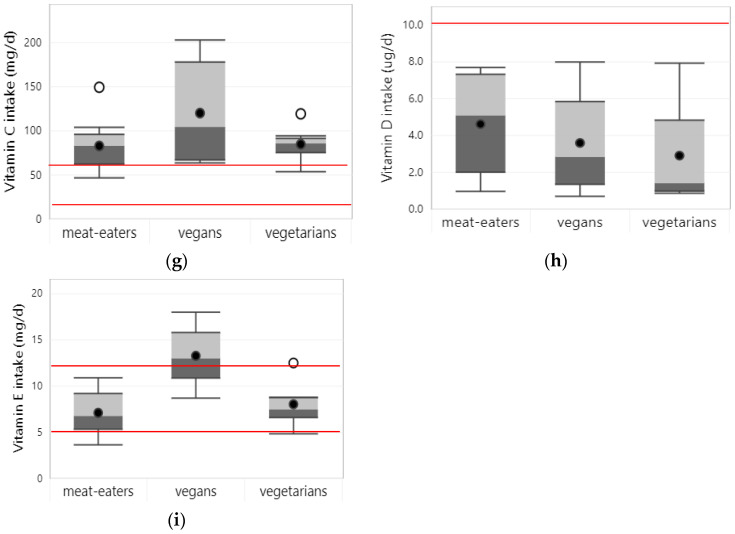
Boxplots represent 25th, 50th, and 75th percentiles of vitamin intakes from foods only, with whiskers at the <1.5 interquartile range (IQR). Black dots represent mean intake and white dots outliers at the >1.5 IQR: (**a**) vitamin A; (**b**) vitamin B1; (**c**) vitamin B2; (**d**) vitamin B6; (**e**) folate; (**f**) vitamin B12; (**g**) vitamin C; (**h**) vitamin D; (**i**) vitamin E. Straight red lines represent the estimated average requirement (EAR) for the age and gender groups with the lowest and highest dietary requirements that were included in the studies. Boxplots are only shown for nutrients, for which intake data from at least 3 studies were available for at least one of the dietary patterns.

Ten studies reported on vitamin A intake, out of which nine assessed intake from foods alone. Two studies, which reported vitamin A intake in international units or retinol activity equivalents, could not be included in the calculation of descriptive intake values [27,31]. Across the remainder of the studies that reported vitamin A intake in µ REg/d (n = 7), average vitamin A intake from foods was higher in vegetarian children and adolescents (1550 µg RE/d) compared to vegans (1002 µg RE/d) and meat eaters (906 µg RE/d) (see Figure 3a). When considering only studies that assessed intakes from foods and supplements, vitamin A intakes were similar between dietary patterns. Across all studies and all dietary patterns, vitamin A intake from foods was well above the EAR of the respective age and gender groups.

Most studies (i.e., 4/6 studies) that compared vitamin A intake from foods between dietary patterns reported no significant differences. The other two studies found significantly higher intakes in vegan and semi-vegetarian as compared to meat-eating children and adolescents in Ghana and the USA [23,31]. Three studies that assessed intakes from foods and supplements found no differences in vitamin A intake between dietary patterns.

Two studies reported on the vitamin A status of children in Poland and the UK, with average retinol levels of vegetarians and meat eaters within the physiological range (i.e., >0.7 µmol/L). The UK study showed significantly higher retinol levels in vegetarian as compared to meat-eating children [26], whereas the Polish study found no significant difference [38]. One additional study among Indian pre-school children assessed vitamin A deficiency based on ocular signs and symptoms, finding a significantly higher prevalence of deficiency in vegetarians (7.1%) compared to meat eaters (1.4%) [24].

#### 3.3.2. Vitamin B1

Eight studies reported on vitamin B1 intake in children and adolescents, out of which six reported on intake from foods alone. Average vitamin B1 intake from foods was the highest in vegans (1.3 mg/d), followed by meat-eaters (1.1 mg/d) and vegetarians (0.7 mg/d). Across studies, vitamin B1 intake from foods generally met the EAR of the respective age and gender groups in all dietary patterns, except for one study among vegetarian school children in China [35].

Four out of five studies showed that vegan children and adolescents had significantly higher vitamin B1 intake from foods than meat eaters. Comparisons between vegetarians and meat eaters were inconsistent, with one study showing significantly higher intake, one study showing significantly lower intake, and one study showing no significant difference in intake. When intake from foods and supplements was considered, most (i.e., 3/4) studies found no difference in vitamin B1 intake between vegetarians and meat eaters, whereas vegans had a significantly higher intake (in 2/2 studies).

#### 3.3.3. Vitamin B2

Eight studies reported on vitamin B2 intake, out of which six reported on intake from foods alone. Among these studies, meat-eating children and adolescents had the highest average vitamin B2 intake from foods (1.4 mg/d). The intake of vegetarians (0.9 mg/d) and vegans was similar (1.0 mg/d). For meat eaters, vitamin B2 intake from foods met the EAR of the respective age and gender groups in all studies; for vegetarians (i.e., 3/4 studies) and vegans (i.e., 4/5 studies), vitamin B2 intake was above the EAR in most studies.

Most studies that compared vitamin B2 intake from foods between dietary patterns found significantly lower intakes in vegans (i.e., 3/5 studies) and vegetarians (i.e., 2/5 studies) compared to meat eaters; the others showed no significant difference. However, when intake from foods and supplements was considered, most (i.e., 3/4) studies found no difference in vitamin B2 intake between vegetarians and meat eaters. For vegans, the results were mixed, with one study showing a significantly higher intake [27] and one study showing a significantly lower intake [29] of vitamin B2 compared to meat eaters. One Ghanese study reported a similar prevalence of children with vitamin B2 intake below the DRV in vegans and meat eaters [23].

No studies reported on vitamin B2 status or deficiency in children or adolescents.

#### 3.3.4. Niacin

Three studies reported on niacin intake in children or adolescents from Finland, Sweden, and the UK [26,27,39], two of which assessed intake from foods alone. In all studies, niacin intake across dietary patterns was well above the EAR of the respective age and gender groups. Out of two studies that compared niacin intake from foods between dietary patterns, one found significantly lower intake in vegans compared to meat eaters [39]; the other found no significant difference in the intake of vegan and vegetarian children and adolescents compared to meat eaters [27]. Two studies that included intake from foods and supplements found similar [27] or significantly lower niacin intake [26] in vegetarians but a significantly higher niacin intake in vegans compared to meat eaters [27].

No studies reported on the prevalence of inadequate niacin intake or niacin status in children or adolescents.

#### 3.3.5. Vitamin B6

Five studies reported on vitamin B6 intake in children and adolescents, all of which assessed intake from foods alone. Average vitamin B6 intake from foods was higher in vegans (1.7 mg/d) and meat eaters (1.6 mg/d) compared to vegetarians (0.9 mg/d). In all studies, vitamin B6 intake from foods was above the EAR of the respective age and gender groups for all dietary patterns.

Most studies (i.e., 4/5) that compared vitamin B6 intake from foods between dietary patterns generally found no significant differences. Only one study among German infants and young children found that vegans had a significantly higher vitamin B6 intake compared to meat eaters and vegetarians [29]. Similar results on vitamin B6 intake were found in two studies that assessed intake from foods and supplements [27,29].

No studies reported on the prevalence of inadequate vitamin B6 intake, vitamin B6 status, or deficiency.

#### 3.3.6. Folate

Ten studies reported on folate intake, of which eight assessed intake from foods alone. Across studies that assessed intake from foods alone, average folate intake was highest in vegans (362 µg/d), followed by vegetarians (247 µg/d) and meat eaters (199 µg/d). In meat eaters, folate intake from foods was below the EAR of the respective age and gender groups in most (i.e., 5/7) studies. For (semi-)vegetarians and vegans, folate intake from foods exceeded the EAR in most (i.e., 6/7) or all studies, respectively.

Studies that compared folate intake from foods between dietary patterns consistently reported a significantly higher intake in vegans compared to meat eaters (i.e., 4/4 studies). (Semi-)vegetarians had a significantly higher intake compared to meat eaters in half of the studies (i.e., 3/6), whereas the other half of the studies showed no significant difference. Similar results were found in studies that assessed intake from foods and supplements. No studies reported on the prevalence of inadequate folate intake.

**Figure 4:**
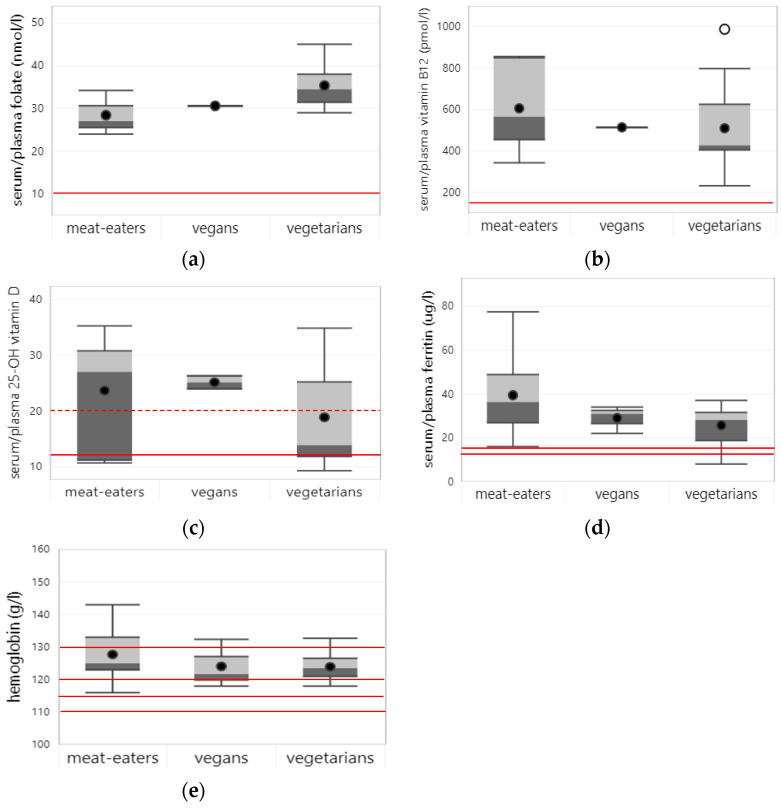
Boxplots represent 25th, 50th, and 75th percentiles of micronutrient status (**a**–**d**) and hemoglobin (**e**) concentrations per dietary pattern based on all studies (i.e., including and excluding supplement users) with whiskers at the <1.5 interquartile range (IQR). Black dots represent mean biomarker concentrations and white dots outliers at the > 1.5 IQR. Straight red lines represent cut-off levels to indicate nutrient deficiency and anemia, i.e., folate (<10 nmol/L), vitamin B12 (<148 pmol/L), vitamin D (<12 µg/L), and ferritin (<12 µg/L for children < 5 y; <15 µg/L for older children). For anemia, cut-off values were 110 g/L for children < 6–59 months, 115 g/L for children 5–11 y, 120 g/L for children 12–14 y and girls 15 y and older, and 130 g/L for boys 15 y and older. The dotted red line represents the cut-off value for vitamin D insufficiency (<20 µg/L). Boxplots are only shown for nutrients, for which status data from at least 3 studies were available for at least one of the dietary patterns.

Eight studies reported on the folate status of children and adolescents, none of which excluded supplement users. Average serum/plasma folate status was highest in vegetarians (35.3 nmol/L), followed by vegans (30.6 nmol/L) and meat eaters (28.1 nmol/L) (see Figure 4a). Studies that directly compared folate status between dietary patterns (n = 6) found similar or higher folate status among (semi-)vegetarian and vegan than in meat-eating children and adolescents. No studies reported on the prevalence of folate deficiencies using established cut-off values.

#### 3.3.7. Vitamin B12

Thirteen studies reported on vitamin B12 intake, out of which ten assessed intakes from foods alone. Across studies that assessed intake from foods alone, average vitamin B12 intake was highest in meat eaters (3.49 µg/d), with considerably lower intake in vegetarian (1.66 µg/d) and vegan (0.59 µg/d) children and adolescents. However, when considering studies that assessed intake from foods and supplements, this pattern reversed, with much higher average vitamin B12 intake among vegans (116.6 µg/d) compared to meat eaters (28.8 µg/d) and vegetarians (5.2 µg/d).

Studies that compared vitamin B12 intake from foods between dietary patterns (n = 9) found that vegetarians had similar (i.e., 3/6 studies) or significantly lower intakes (i.e., 3/6 studies) compared to meat eaters, whereas vegans had significantly lower intake in most (i.e., 3/4) studies. On the contrary, studies that considered intake from foods and supplements (n = 4) showed that the vitamin B12 intake of vegan children was significantly higher (in 2/2 studies), whereas for vegetarian children it was similar compared to that of meat-eating children in most (i.e., 3/4) studies.

For meat eaters, vitamin B12 intake from foods met the EAR of the respective age-and gender groups in all studies. Among (semi-)vegetarians, most (i.e., 4/6) studies also found vitamin B12 intake from foods above the EAR, whereas vegans mostly had an intake below the EAR (i.e., 4/5 studies). Notably, studies that assessed intake from foods and supplements (n = 5), generally found that vitamin B12 intake was above the EAR for all dietary patterns, except for one study among vegetarian children in Taiwan [40].

Nine studies reported on vitamin B12 status, one of which excluded supplement users. Across all studies, average vitamin B12 serum/plasma levels were somewhat higher in meat-eating (564 pmol/L) compared to vegan (514 pmol/L) and vegetarian (510 pmol/L) children and adolescents. The lowest vitamin B12 levels (232 pmol/L), which were still well within the physiological range, were reported in a study among vegetarian Indian immigrant girls in New Zealand, which excluded supplement users [41].

Studies that compared vitamin B12 status across dietary patterns found mixed results. Among six studies that included supplement users, four showed no significant difference in vitamin B12 status between vegetarians or vegans and meat eaters; the other two studies reported significantly lower vitamin B12 status in semi-vegetarians compared to meat-eating children and adolescents (see Figure 4b). Two studies that excluded supplement users also found significantly lower vitamin B12 status in vegetarians and vegans compared to meat eaters [41,42].

One study reported on vitamin B12 deficiency (i.e., serum vitamin B12 < 148 pmol/L) in Polish school children [42]. The prevalence of vitamin B12 deficiency was low in meat eaters and vegetarians (3.2% and 3.8%) but significantly higher in vegans (13%).

#### 3.3.8. Vitamin C

Eleven studies reported on vitamin C intake in children and adolescents, of which nine reported on intake from foods alone. Average vitamin C intake from foods was higher in vegan (120 mg/d) compared to vegetarian (85 mg/d) and meat-eating (83 mg/d) children and adolescents. Across dietary patterns, vitamin C intake from foods met the EAR for the respective age and gender groups in all studies. Studies that compared vitamin C intake from foods between dietary patterns (n = 8) found that both vegetarians and vegans had similar (in 3/5 and 2/5 studies, respectively) or significantly higher intake (in 2/5 and 3/5 studies, respectively) compared to meat-eating children and adolescents. Semi-vegetarian children and adolescents in the USA had similar vitamin C intake as meat eaters [31]. Studies that assessed vitamin C intake from foods and supplements (n = 4) showed comparable results. Two studies reported on the prevalence of children with vitamin C intakes below the DRI in the UK and Ghana, with no significant differences between dietary patterns [23,26].

One study investigated vitamin C status in vegetarian and meat-eating young children in the UK. Average vitamin C plasma levels were adequate (68–88 µmol/L), with no significant difference between dietary patterns [26].

#### 3.3.9. Vitamin D

Seven studies reported on vitamin D intake, of which five assessed intake from foods alone. Vitamin D intake from foods was highest in meat-eating children and adolescents (4.62 µg/d), followed by vegetarians (3.60 µg/d) and vegans (2.91 µg/d). In all of the studies and across all dietary patterns, vitamin D intake from foods was below the EAR of the respective age and gender groups (i.e., <10 µg/d) [21]. In addition, studies that assessed vitamin D intake from foods and supplements (n = 5) generally reported intakes below the EAR, except for young vegan children in Germany [29] and vegan, vegetarian, and meat-eating children in Finland [27].

Of the studies that compared vitamin D intake from foods alone (n = 4) or from foods and supplements (n = 4), half found significantly lower intakes in vegetarians and vegans compared to meat-eating children and adolescents, whereas the other half found no differences in vitamin D intake. The prevalence of vitamin D intake below the DRV, as reported in two studies from the UK and Poland, was high across all dietary patterns, irrespective of whether intake from supplements was considered [26,43].

Six studies reported on vitamin D status in children and adolescents, none of which excluded supplement users. Across all studies, average 25-OH vitamin D levels were higher in vegans (25.2 µg/L) and meat eaters (23.7 µg/L) compared to those of vegetarians (18.9 µg/L) (see Figure 4c). Average 25-OH vitamin D levels of vegetarians did not meet the cut-off value for adequate vitamin D status (i.e., <2.0 µg/L [21]), suggesting a high risk of vitamin D insufficiency among vegetarian children. The lowest 25-OH vitamin D levels were reported in a study among young children in the UK, which found mean 25-OH vitamin D levels for both vegetarian and meat-eating children below the cut-off value for vitamin D deficiency [44]. No studies reported on the prevalence of vitamin D deficiency, but one study from Germany reported on vitamin D insufficiency (i.e., <20 µg/L), which was present in 36% of vegetarian, 27% of vegan, and 28% of meat-eating children and adolescents.

Studies that compared vitamin D status between dietary patterns found significantly lower or similar intake in vegans as well as vegetarians compared to meat-eating children and adolescents.

#### 3.3.10. Vitamin E

Eight studies reported on vitamin E intake in children and adolescents, of which six assessed intake from foods alone. Average vitamin E intake from foods was similar in vegetarians (8.0 mg/d) and meat eaters (7.1 mg/d) but was clearly higher in vegans (13.3 mg/d). Among vegans, average vitamin E intake from foods met the EAR of the respective age and gender groups in all studies. For meat eaters and vegetarians, average intake fell below the EAR in two and one out of five studies, respectively. Four out of five studies that compared vitamin E intake from foods between dietary patterns found significantly higher intake in vegans and/or vegetarians compared to meat-eating children and adolescents. Similar results were found in studies that assessed intake from foods and supplements (n = 4). No studies reported on the prevalence of inadequate vitamin E intake.

Two studies reported on vitamin E status in young and school-age children in Poland and the UK, with average a-tocopherol levels within the physiological range (>12 µmol/L). In vegetarian children in the UK, vitamin E status was significantly higher than or similar to meat eaters [26], whereas in Polish vegetarian children vitamin E status was significantly lower [38]. No studies assessed the prevalence of vitamin E deficiency.

### 3.4. Minerals

#### 3.4.1. Iron

**Figure 5:**
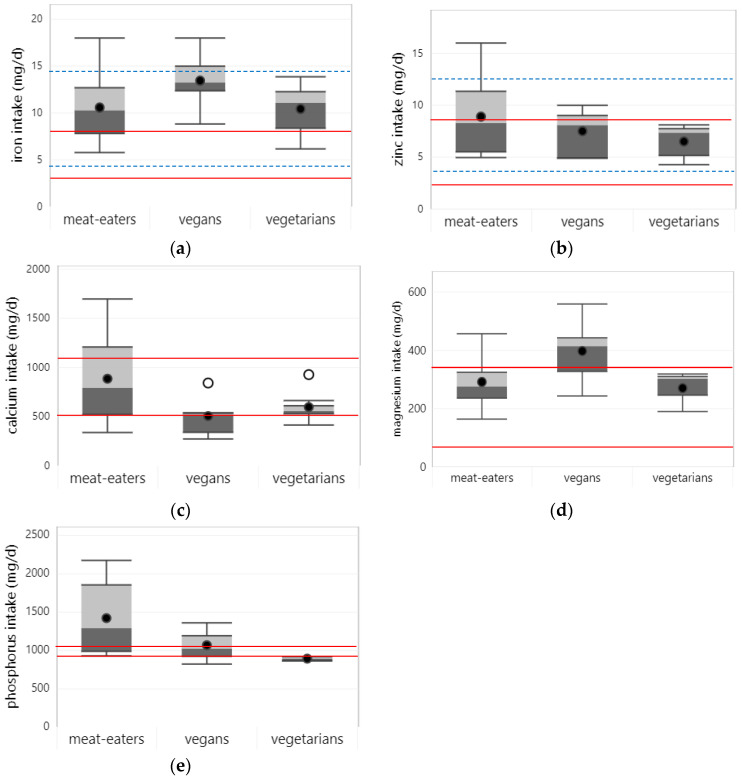
Boxplots represent 25th, 50th, and 75th percentiles of mineral intake based on studies that assessed intake from foods only, with whiskers at the <1.5 interquartile range (IQR). Black dots represent mean intake and white dots outliers at the >1.5 IQR: (**a**) iron; (**b**) zinc; (**c**) calcium; (**d**) magnesium; (**e**) phosphorus. Straight red lines represent the estimated average requirement (EAR) for the age and gender groups with the lowest and highest dietary requirements that were included in the studies. Dotted blue lines represent bioavailability-adjusted EAR for iron and zinc for the age and gender groups with the lowest and highest dietary requirements. Boxplots are only shown for nutrients, for which status data from at least 3 studies were available for at least one of the dietary patterns.

Twelve studies reported on iron intake in children and adolescents, of which nine assessed intake from foods alone. On average, the highest iron intake from foods was observed in vegans (13.5 mg/d); the intake of vegetarians and meat eaters was similar (10.5 and 10.6 mg/d) (see Figure 5a). Across dietary patterns, iron intake from foods generally met the EAR for the respective age and gender groups in all studies. However, when adjusting the EAR for the lower bioavailability of plant-based diets, iron intake from foods was below the EAR in one out of six studies among vegetarians and one out of five studies among vegan children and adolescents. Iron intake from foods and supplements, as reported in five studies, generally met the (bioavailability-adjusted) EAR across all dietary patterns, except for one study that reported a lower intake among vegetarian adolescent girls in Poland [45].

Of the eight studies that compared iron intake from foods between dietary patterns, half reported higher iron intake in vegetarian and/or vegan children and adolescents compared to meat eaters, whereas the others showed no significant difference. Similar results were found in studies that included intake from supplements (n = 5). Three studies that assessed the prevalence of iron intake below the DRV did not show differences between dietary patterns.

Eleven studies reported on the iron status of children and adolescents, one of which excluded supplement users. Across all studies, average ferritin levels were higher in meat-eating children and adolescents (39.5 µg/L) compared to vegans (29.0 µg/L) and vegetarians (25.7 µg/L). Reported ferritin levels were mostly within the physiological range for all dietary patterns, except for one study from the UK that reported low ferritin levels in 1–3-year-old vegetarian children [26].

Seven studies assessed the prevalence of iron deficiency, i.e., defined as serum ferritin < 12 µg/L in children < 5 years or <15 µg/L in children ≥ 5 years. The average prevalence of iron deficiency was higher in vegetarians (28.1%) and vegans (20.7%) compared to meat-eating children and adolescents (14.1%). Prevalence numbers varied a lot, with the highest iron deficiency prevalence reported in 1–3-year-old vegetarian children (73%) and vegetarian adolescent girls (53%), both in the UK [26,46].

Most studies (i.e., nine out of ten) that compared iron status between dietary patterns found significantly lower ferritin levels in vegetarians and vegans compared to meat-eating children and adolescents (see Figure 4d). One of these studies found significantly lower iron status in vegetarian adolescent girls only but not in vegetarian boys [47].

Nine studies also measured hemoglobin, with slightly higher values on average in meat-eating children and adolescents (128 g/L) than in vegetarians (124 g/L) and vegans (124 g/L). However, unlike for serum ferritin, the studies did not show significant differences in hemoglobin levels between dietary patterns (see Figure 4e). The average prevalence of anemia was similar across dietary patterns, with 13.8%, 15.5%, and 15.2% in meat eaters, vegetarians, and vegans, respectively. The highest anemia prevalence was observed in vegetarian and meat-eating children and adolescents in India (30–45%) [47].

#### 3.4.2. Zinc

Ten studies reported on zinc intake in children and adolescents, of which eight assessed intake from foods alone. Among these studies, average zinc intake from foods was somewhat higher in meat eaters (8.9 mg/d) compared to vegetarians (7.5 mg/d) and vegans (6.5 mg/d). Across all dietary patterns, zinc intake from foods met the EAR of the respective age and gender groups in all but one Polish study, where zinc intake fell short in both vegetarian and meat-eating children [48]. When the EAR was adjusted for the lower bioavailability of plant-based diets, the adequacy of zinc intake decreased among (semi-)vegetarians and vegans, with mean zinc intake from foods below the EAR in three out of six and two out of five studies, respectively. However, studies that considered intake from foods and supplements (n = 4) all reported mean intakes above the bioavailability-adjusted EAR for vegetarians and vegans. Two studies reported on the prevalence of zinc intakes below the DRV among young and school-age children in the UK and Ghana, with no significant differences between dietary patterns [23,26]. Across all dietary patterns, zinc intake from foods met the EAR of the respective age and gender groups in all but one Polish study, where zinc intake fell short in both vegetarian and meat-eating children [48]. When the EAR was adjusted for the lower bioavailability of plant-based diets, the adequacy of zinc intakes decreased among (semi-)vegetarians and vegans, with mean zinc intake from foods below the EAR in three out of six and two out of five studies, respectively. However, studies that considered intake from foods and supplements (n = 4) all reported mean intakes above the bioavailability-adjusted EAR for vegetarians and vegans. Two studies reported on the prevalence of zinc intake below the DRV among young and school-age children in the UK and Ghana, with no significant differences between dietary patterns [23,26]. Most (i.e., 8/9) studies also found no significant difference in zinc intake from foods alone or from foods and supplements between dietary patterns, except for one study among Swedish adolescents that showed a significantly lower zinc intake from foods in vegans compared to meat eaters [39].

One study assessed plasma zinc concentrations in 1.5–4.5-year-old children from the UK. Average zinc concentrations were within the physiological range (>65 µg/dL), with no significant difference between meat eaters and vegetarians [26]. No studies reported on the prevalence of zinc deficiency in children or adolescents.

#### 3.4.3. Iodine

Two studies reported on iodine intake in children from Germany and Finland [27,29], both assessing intake from foods alone as well as from foods and supplements. Among Finnish 1–7-year-old children, mean iodine intake from foods was above the EAR for all dietary patterns, whereas among 1–3-year-children from Germany, mean intake was below the EAR for all dietary patterns, i.e., vegans, vegetarians, and meat eaters. Iodine intake remained below the EAR, including when intake from supplements was considered. Both studies reported significantly lower iodine intake from foods in vegetarian and vegan compared to meat-eating children. However, when intake from foods and supplements was considered, the difference in iodine intake between dietary patterns disappeared in both studies.

No studies reported on iodine status or the prevalence of iodine deficiency in vegetarian or vegan children and adolescents.

#### 3.4.4. Calcium

Eleven studies reported on calcium intake in children and adolescents, of which eight reported on intake from foods alone. The average calcium intake from foods was highest in meat eaters (886 mg/d), followed by vegetarian (598 mg/d) and vegan (506 mg/d) children and adolescents. Across studies that assessed calcium intake from foods and supplements (n = 5), average calcium intake was comparable between dietary patterns (596–662 mg/d). In most (i.e., 6/8) studies, calcium intake from foods was below the EAR of the respective age and gender groups for all dietary patterns. In one study, zinc intake was below the EAR for vegans but not for meat-eating children [39]. Only one study among Finnish children found mean calcium intake from food above the EAR for all dietary patterns (i.e., vegans, vegetarians, and meat eaters) [27]. In addition, when considering intake from foods and supplements, most (i.e., three out of five) studies still found mean calcium intake below the EAR for all dietary patterns.

Studies that compared calcium intake between dietary patterns from food alone or from foods and supplements (n = 10) generally found no significant difference between vegetarian and meat-eating children or adolescents (in 6/7 studies); vegans had similar (in 2/5 studies) or significantly lower intakes (in 3/5 studies) compared to meat eaters. In addition, four studies that assessed the prevalence of children and adolescents with calcium intakes below the DRV found no significant differences between dietary patterns.

Three studies assessed bone turnover markers and one study also assessed bone mineral density in vegetarian and meat-eating children from Poland. The results on bone turnover markers were mixed. Two studies found significantly lower levels of bone formation (i.e., osteocalcin, bone alkaline phosphatase (BAP)) and bone resorption markers (i.e., collagen type I terminal telopeptide (CTX)) in vegetarian children compared to meat eaters, indicating an impaired bone turnover rate in vegetarians. The other study reported significantly higher levels of BAP as well as CTX in vegetarian children, suggesting an increased rate of bone turnover [43]. The latter study also reported no significant difference in bone mineral density between vegetarians and meat eaters [43].

#### 3.4.5. Magnesium

Four studies reported on magnesium intake in children and adolescents, all of which assessed intake from foods alone. In all studies and for all dietary patters, mean intake was above the EAR of the respective age and gender groups. The average magnesium intake from foods alone was higher in vegans (398 mg/d) compared to meat-eating (292 mg/d) and vegetarian (271 mg/d) children and adolescents. Four studies that compared magnesium intake between dietary patterns consistently found significantly higher intake in vegans and vegetarians than in meat-eating children and adolescents.

No studies reported on the prevalence of inadequate magnesium intake, magnesium status, or deficiency.

#### 3.4.6. Phosphorus

Four studies reported on phosphorus intake, of which three assessed intake from foods alone. Average phosphorus intake from foods was higher in meat eaters (1423 mg/d) compared to vegetarian (1070 mg/d) and vegan children and adolescents (894 mg/d). In all studies, phosphorus intake of meat-eating and vegetarian children and adolescents met the EAR of the respective age and gender groups, and no significant differences in phosphorus intake were found between vegetarians and meat eaters in any of the studies that compared intake between dietary patterns (n = 4). However, one of two studies among vegans reported mean phosphorus intake from foods below the EAR in adolescent Swedish girls. Both studies also reported a significantly lower phosphorus intake in vegans compared to meat eaters, including when intake from foods and supplements was considered.

One Polish study reported on phosphorus status, with no significant differences between vegetarian and meat-eating children [49]. No studies assessed the prevalence of inadequate phosphorus intake or phosphorus deficiency.

## 4. Discussion

### 4.1. Main Findings and Their Significance

The outcomes of this comprehensive overview on the nutrient intake and status of children following a predominantly plant-based diet compared to those following a dietary pattern containing meat are summarized in Table 2. No significant differences in energy intake were found between studies that compared plant-based and meat-containing diets. For macronutrients, mean protein intake was generally within recommendations in all diet groups but lower in children consuming plant-based diets, especially in vegans. Fiber, SAFA, and PUFA intakes were highest in vegan children, with average intakes generally meeting recommendations, and lowest in meat-eating children, with average intakes not meeting the recommendations in any of the studies. In addition, vegetarian children seem to be at risk of inadequate fiber, SAFA, and possibly PUFA intakes, but these intakes were more favorable compared to those of meat-eating children. Limited data on omega-3 fatty acid intake from only two studies indicate that EPA and DHA intake may be suboptimal in all diet groups.

For micronutrients, mean vitamin D and calcium intakes were inadequate in all diet groups, and limited data of two studies also indicate a risk of inadequate iodine intake across all diet groups. In addition, children consuming meat may be at risk of inadequate folate and vitamin E intake, whereas children consuming plant-based diets may risk inadequate vitamin B12, iron, and zinc intakes. Mean vitamin A, vitamin B1, vitamin B2, niacin, vitamin B6, vitamin C, and magnesium intakes were adequate in all diet groups.

### 4.2. Strengths and Limitations of This Review

This is the first systematic review that aims to quantify differences in nutrient intake and status between plant-based diets and diets containing meat in children and adolescents aged 2–18 y. We applied common definitions for the different dietary patterns (semi-vegetarian, vegetarian, vegan, and diets containing meat) across all studies to ensure a consistent interpretation of the data. Another strength is that for comparability and consistency, we only included studies that used biomarkers and cut-off levels as applied by the IOM and WHO to assess the prevalence of nutritional deficiencies. In addition, with the restriction of including only studies published between 2000 and 2022 and 17 out of 30 included studies having been published after 2010, our findings are more reflective of the current situation than earlier reviews [11,12]. The outcomes of our review provide insights into possible nutritional risks in different diets of children and adolescents, which may help to develop practical guidance that can help families with children to transition to more healthy and sustainable diets.

A major limitation is that we could not provide reliable estimations on the adequacy of dietary nutrient intake. To evaluate the adequacy of dietary intake in a population, ideally the proportion of the population with usual intakes below the EAR should be determined [50]. However, most studies did not provide this information. Therefore, we compared mean or median intakes with the EAR (or the lower bound of the AMDR) to indicate (in)adequacy of nutrient intakes in the population. It can be assumed that if the mean intake is at or below this level, a substantial proportion of the population will have an intake less than the requirement and is therefore at risk of deficiency. For ALA and fiber, for which only an adequate intake (AI) level is available (i.e., based on the nutrient intake of a group of healthy people, who are assumed to be adequate), it can only be assumed that the prevalence of inadequate intake is low when the mean intake is above the AI [50]. In addition, as many studies reported data for a specific age range, it was not possible to disentangle nutrient intake data for the different age groups as defined by WHO/FAO recommendations and benchmark these data to their corresponding EARs. Therefore, for these studies, we chose to benchmark mean intake data against the dietary reference value for the mean age of the children included in the study. This may have led to further inaccuracies in the estimates of (in)adequate intakes.

Based on the risk of bias assessment, the quality of the included studies was mixed. For many studies (n = 14), the representativeness of the study sample was not clear or not considered optimal because the children following a plant-based diet had been referred to the research institute for dietary counseling or because they were recruited from specific religious communities. Hardly any study (n = 4) reported to have performed a power calculation or otherwise justified a sufficient sample size, and most studies (n = 19) were relatively small (i.e., <50 subjects per dietary pattern). In addition, most studies (n = 20) did not control their analysis for potential confounders.

Overall, there were little data for non-Western populations, as 80% of the studies (24 out of 30) were conducted in high-income Western countries, mainly in European countries. Therefore, our results are mostly representative of Western populations.

### 4.3. Putting Findings on Energy and Macronutrients into Perspective

Whereas some studies have suggested that energy intake in children consuming plant-based and, in particular, vegan diets is lower compared to that of meat eaters, our review did not find any such differences. Moreover, previous reviews evaluating the physical growth of children following vegetarian and vegan diets found that they had normal weights [11,12,16], suggesting that plant-based diets contain sufficient energy for healthy growth and development. Whereas our review showed that across studies, average protein intake was lower but adequate in children consuming plant-based diets, data on protein quality are lacking. There is debate on whether plant protein would be of sufficient quality to meet the needs for the healthy growth of children and whether dairy would be essential for optimal growth. However, the biological basis for the positive effect of milk on growth is unknown; it may be related to high protein quality or the presence of calcium, phosphorus and magnesium, lactose, or specific amino acids, which may have growth-promoting abilities or other unknown factors [51,52]. Dairy intake was associated with a higher diet quality in two studies, i.e., one in Australia [53] and one in multiple countries in Europe [54]. A recent systematic review in children 3–18 y showed that dairy consumption is associated with higher bone mineral density but that the relationship with linear growth is inconclusive [55]. With the shift to more plant-based diets, it would be of interest to investigate whether dairy consumption would have a benefit over plant-based diets without dairy that are nutritionally adequate with respect to protein quality and micronutrients needed for bone density and linear growth.

The higher intake of fiber and PUFA and the lower intake of SAFA in vegan children and adolescents can be explained by the exclusive intake of plant foods and was in contrast with the unfavorable intakes of fiber, PUFA, and SAFA in vegetarian and meat-eating children, which were generally not in line with the recommendations. This indicates that children’s intake of whole grains, vegetables, fruits, and nuts could generally be improved. The limited data showing that the longer-chain omega-3 fatty acids EPA and DHA were below the recommendations are in line with findings of previous studies on the general population of children in Europe [56] and can be explained by very low fish and seafood consumption.

### 4.4. Putting Findings on Micronutrients into Perspective

For micronutrients, vitamin A, vitamin B1, vitamin B2, niacin, vitamin B6, vitamin C, and magnesium studies generally showed adequate average intakes (i.e., >EAR) in all diet groups. Vitamin A intake was well above the EAR for all diet groups, but these estimates may have been too high, as for some studies it was unclear from the publications whether and how vitamin A from carotenoids present in plant foods was converted to retinol equivalents. Moreover, in most studies, intake was expressed in RE, as recommended by EFSA, which means that a factor of 6 is used to convert the intake of carotenoids to vitamin A equivalents, whereas IOM expresses vitamin A intake in RAE by using a conversion factor of 12, which then results in lower intake levels [20]. When children depend on plant foods for vitamin A intake, it is important to include vitamin A-rich fruits and vegetables in the diet, such as pumpkin, carrots, and mangos.

Micronutrients that are highly present in plant foods (folate, magnesium, and vitamins C and E) were markedly higher in the diets of vegans compared to the diets of meat eaters, based on expectations, whereas this difference was much smaller for vegetarians compared to meat eaters. This indicates that the intake of plant foods among vegetarian and meat-eating children is more similar, which is also reflected in the results for fiber.

We generally lack data to fully conclude whether the intake of micronutrients is adequate to prevent deficiencies, except for folate, vitamin B12, vitamin D, and iron. For iron, dietary intake seemed adequate for all diet groups, including when taking the lower bioavailability of 10% of iron in plant foods into account. However, the prevalence of iron deficiency was substantially higher in vegetarian and vegan children compared to meat-eating children, which shows that there is still a greater risk of inadequate iron intake when consuming plant-based diets. For zinc, the intake of children consuming plant-based diets was below the bioavailability-adjusted EAR in roughly half of the studies, and data on zinc status were too limited (with only one study) to confirm these findings. We therefore recommend that future studies on children and adolescents include measurements of micronutrient status. In addition, further research should clarify which recommendations for lower bioavailable iron and zinc content in plant-based diets are applicable to meet the physiological needs of children.

Furthermore, the data from our review are generally too limited to draw firm conclusions on whether supplementation can help meet micronutrient requirements, as the studies often did not mention whether nutrient intakes from supplements were considered. We therefore assumed that intake data in these studies were based on intake from foods only, whereas some studies may still have included intakes from supplements. This could have led to an overestimation of nutrient intakes from foods and blurred the difference in nutrient intakes from foods with supplements. Furthermore, heterogeneity in the proportion of supplement users across studies, as well as the dose and type of vitamins and minerals used, may have blurred the findings. However, in studies that reported intakes from food and supplements, it was shown that supplementation with iron, zinc, and vitamin B12 could help achieve the (higher) recommended intakes in the children consuming plant-based diets, whereas this was not the case for studies on calcium and vitamin D supplementation. These findings indicate that it will be important to educate parents and caregivers on the need for supplementation when required amounts of micronutrients cannot be met by the diet.

Lastly, it is important to mention that from the included studies it was not clear to what extent additional micronutrient intake from fortified foods was considered, which may have led to an underestimation of nutrient intakes in our overall findings.

### 4.5. Findings in Other Age Groups

Two similar reviews, both of which also included studies published before 2000, evaluated nutrient intake in plant-based versus omnivorous diets in children [11,12]. Sutter et al. [11] reviewed data from six studies on vegan children aged 0–10 y and found similar results for energy, protein, iron, calcium, folate, and vitamins B12 and D. Schürmann et al. [12] performed a systematic review including 16 studies on vegetarian children 0–18 y from industrialized countries and had similar findings for fiber, folate, and vitamins B12, C, and D. Unfortunately, data from our review did not allow for evaluation of whether the nutritional adequacy of the diets differs between children and adolescents, because the studies often provided combined data for children and adolescents and few studies (n = 5) reported on adolescents alone. However, adolescent girls may, in general, have a higher risk of iron deficiency and anemia due to additional needs for menstruation. We previously reviewed the energy and nutrient intake and status of adults consuming plant-based diets versus adults consuming meat [17] and found similar results. Risk of inadequate iron intake was particularly higher for women of reproductive age. This means that currently available data indicate that the risks of nutrient inadequacies are similar in children, adolescents, and adults and are dependent on the diet type rather than age-related factors.

We did not include studies conducted on children younger than two years of age, as these infants and young children have specific dietary requirements and need breastfeeding. A recent review evaluated the evidence on the impact of vegan and vegetarian complementary feeding on the nutrient intake, growth, and neurodevelopment of infants [57]. The studies were very limited, but similar to our findings for children and adolescents, the authors concluded that vegetarian and vegan children aged 6–24 months are at risk of iron, vitamin B12, and vitamin D deficiencies and that appropriate supplementation and dietary guidance are needed.

### 4.6. Public Health Recommendations

Currently, health authorities generally state that well-planned vegetarian diets can meet the nutritional needs of children and adolescents, but opinions about the appropriateness of vegan diets are controversial. Health authorities in the USA, Canada, the UK, Portugal, and Australia are of the opinion that vegetarian and vegan diets can be suitable for children when they are well planned and adequately supplemented [58,59,60,61,62], whereas in France, Germany, and Spain, health authorities do not recommend vegan diets for children and adolescents [63,64,65] or children under the age of two years [58]. In addition, most health authorities indicate that medical and dietetic supervision is strongly recommended when children consume vegan diets [60,63,64,65,66].

The findings of the current paper confirm that, not only for vegan or vegetarian children but regardless of diet type, parents and caregivers would benefit from dietary counseling to improve diet quality to ensure it contains all of the nutrients needed for the healthy growth and development of their children. Specifically, calcium, vitamin D, and iodine intake could be improved across all diet groups, as well as fat quality and fiber intake in both vegetarian and meat-eating children. For children following a plant-based diet, additional focus needs to be placed on sufficient intake of iron, zinc, and vitamin B12, whereas for meat-eating children, intake of folate and vitamin E should be improved.

For diets, our findings indicate that meat-eating and vegetarian children in particular should consume more plant-based foods, like whole grains, legumes and pulses, vegetables and fruits, and nuts and seeds, to achieve adequate intakes of fiber, SAFA, PUFA, folate, and vitamin E. When no or lower amounts of animal foods are consumed, it is essential that children consume a large variety of nutrient-dense plant foods (i.e., high in iron, zinc, iodine, calcium, and vitamins B12 and D) and/or foods, e.g., that are appropriately fortified with these micronutrients, e.g., dairy and meat alternatives. However, when such foods are not available or not consumed in sufficient amounts, supplementation is recommended. This means that public health professionals have a responsibility to provide counseling more actively to parents and provide them with practical materials to offer healthier and more sustainable meals and snacks for their children. In addition, governments have a role to ensure that nutritious plant-based foods, including fruits and vegetables, are affordable and available. Furthermore, industry should continue efforts to improve the nutritional quality of products by lowering sodium, sugar, and SAFA while increasing fiber, PUFA, and micronutrient content.

## 5. Conclusions

This systematic review shows that children on a plant-based diet as well as meat-eating children are at risk of inadequate nutrient intakes. Vitamin D, calcium, and likely also iodine and EPA and DHA seem to be at risk across all diet groups. In addition, the results indicate that children on plant-based diets may be at risk of inadequate intakes of iron, zinc, and vitamin B12, whereas meat-eating children may be at risk of inadequate vitamin E and folate intakes. In contrast, for vegan children, SAFA, PUFA, and fiber intakes were generally not in line with the recommended intakes compared to vegetarian and meat-eating children.

Increasing consumption of a variety of nutrient-rich plant foods, in combination with food fortification and possibly supplementation, can help children and adolescents have more sustainable diets that are healthier and meet all nutrient requirements. However, as research in children is generally limited, future well-designed observational studies are needed to monitor the effects of the different types of plant-based diets on nutrient intake and status, using appropriate biomarkers and investigating more functional outcomes, such as growth, development, and prevention of NCDs. More data are needed, particularly from countries outside Europe, which will allow for the exploration of cultural and geographical differences in the adequacy of plant-based and meat-containing diets.

## Figures and Tables

**Table 1:** Characteristics of the 30 included studies.

Characteristics	Number of Studies (n)
Young children (2–5 y)	15
School children (6–12 y)	24
Adolescents (13–18 y)	11
Meat-eating	27
Vegetarian	26
Vegan	7
Semi-vegetarian	3
Europe	19 (mostly Poland)
Asia	5 (mostly India)
North America	2 (US, Canada)
Oceania	3 (New Zealand, Australia)
Africa	1 (Ghana)
Nutrient intake, assessed from foods alone	14
Nutrient intake, assessed from foods and supplements	8
Nutrient status in users and non-users of supplements	20
Nutrient status in non-users of supplements only	3

**Table 2:** Overview of nutrients at risk of inadequacy and nutrients of favorably high intake across dietary patterns.

Dietary Pattern	Nutrients at Risk of Inadequacy *	Nutrients of Favorably High Intake
Vegans	Vitamin B12, vitamin D	PUFA, fiber
	Calcium, iron, zinc	Vitamin C, vitamin E, folate
Vegetarians	SAFA, PUFA **, fiber	
	Vitamin B12, vitamin D	Vitamin E, folate
	Calcium, iron, zinc	
Meat eaters	SAFA, PUFA, fiber	Vitamin B12
	Vitamin D, vitamin E, folate	Zinc
	Calcium	

* All diet groups may be at risk of inadequate intake of iodine and EPA and DHA, but data are too limited to draw firm conclusions. ** Whereas mean PUFA intake in vegetarian children was in line with the recommendations, the mean intake inadequate in three out of seven studies.

## Data Availability

Data can be made available upon request. A formal data sharing agreement should be signed.

## Supplementary Materials

The following are available online at https://www.mdpi.com/article/10.3390/nu15204341/s1, Table S1: Details of individual studies [67,68,69,70,71,72,73]. Table S2: Summary statistics of nutrient intake data per dietary pattern; Table S3: Summary statistics of nutrient status data per dietary pattern; Table S4: Energy and nutrient intake data per individual study; Table S5: Nutrient status data per individual study; Table S6: Observational study quality evaluation.
